# Development of smart cold forging die life cycle management system based on real-time forging load monitoring

**DOI:** 10.1038/s41598-022-17564-7

**Published:** 2022-08-02

**Authors:** Young Ho Seo

**Affiliations:** Automotive Materials and Components R&D Group, KITECH, Cheomdan-venturero 108, Gwangju, 61007 Korea

**Keywords:** Mechanical engineering, Materials science

## Abstract

Cold forging dies are manufactured through the shrink fit process to withstand high pressure loads, but fatigue failure eventually occurs due to repeated compressive stresses. The life cycle until fatigue failure was defined as the limit life, and attempts were made to predict the die life based on finite element method. However, accurate prediction was impossible owing to uncontrollable environmental variables. Consequently, it is impossible to clearly determine the die replacement cycle, resulting in negative consequences such as poor quality, production delay, and cost increase. Various environmental factors affecting the prediction of die life cycle result in the increase or decrease of the forming load, which is an important variable that determines the die life cycle. In this study, a system for monitoring load data generated from forging facilities was developed based on a piezo sensor. In addition, the die life cycle was more accurately predicted by using the forming load data measured in real time, and a die life management system that can determine the die replacement cycle was applied to the automobile steering parts production line.

## Introduction

The manufacturing industry in modern society is facing various problems due to excessive increase in manufacturing costs, including material and labor costs, rapid demand fluctuations, excessive equipment investment, and surplus production resources^1^. In particular, as the carbon emission regulations are strengthened^2^, the required specifications of the final product are changing in various ways, along with the improvement and innovation of the manufacturing process^3^. To improve the fuel efficiency of automobiles, it is required to reduce the weight of all parts^4^; simultaneously, non-environmental factors must be excluded from the manufacturing process. Consequently, the manufacturing industry was faced with the challenge of simultaneously achieving eco-friendliness, high quality, and low cost. To overcome this situation, efforts are being made to improve the efficiency of the manufacturing process by making various attempts, such as the establishment of a low-cost production structure and the expansion of the automated process. This flow has led to the wave of the fourth industrial revolution that started in Germany^5^ and is accelerating a paradigm shift in the manufacturing industry. Innovation in the manufacturing sector refers to hyper-connection centered on process data and includes analysis and utilization of big data, Internet of Things (IoT), addictive manufacturing, simulation, and horizontal and vertical integration systems^6^.

**Figure 1:**
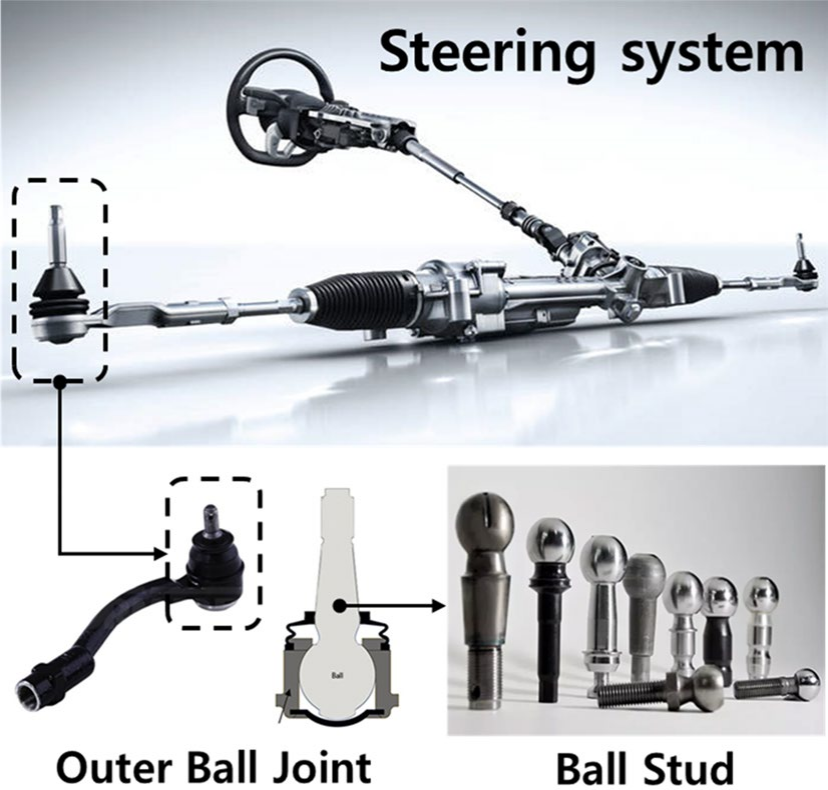
Ball stud parts of a steering system^7–10^.

In this study, as part of the paradigm shift for manufacturing innovation, data from the manufacturing process of automobile steering parts were collected. Based on this, the life span of the forging die was predicted more accurately. Furthermore, it was attempted to maximize the efficiency in the manufacturing process by monitoring the die replacement cycle by the operator. The ball stud in Fig. 1 is connected to the outer ball joint (OBJ), one of the steering systems of automobiles, and plays a role in securing mobility in various directions.

## Literature review

Ball studs are manufactured through a multi-stage cold forging process, in which bulk materials are pressed several times in a closed space to form a final product. Forging operations consist of forming the component by means of plastic deformation of raw material, compressed between a punch and die^11^. In particular, the cold forging process can secure high strength and high shape precision by deforming the material at room temperature^12^. In this process, the die is repeatedly subjected to a high compressive load, and the die material reaches the fatigue limit and becomes damaged^13^. This leads to an increase in process costs^14^, e.g., decrease in productivity and increase in the defect rate due to die fracture and replacement. Studies have been conducted to predict the lifespan of cold forging dies and reduce process costs. The most common way to predict the life of a cold forging die is to use FEM. However, these methods do not quantitatively predict the limiting life, but remain in a qualitative analysis^13,15^. On the other hand, there are studies conducted from the viewpoint that the breakage of the forging die is caused by fatigue cracking^14,16,17^. Tanrıkulu calculated the fatigue limit of the material of the cold forging die and presented an empirical formula to predict the limit life of the die based on the stress value acting on the die through numerical simulation^18^. In addition, similar studies for predicting the life of cold forging dies are continuing^19–23^.

However, in the manufacturing site, the lifespan of the die is still managed based on the experience of the operator, and the breakage of the die occurs suddenly because various working environment variables cannot be considered. There are two main reasons why various research results cannot be applied to the field. The first is that the stress prediction of the die based on simulations does not match the fatigue failure of the die that occurs in the field. Predictions based on finite element method (FEM) is an ideal result that does not take into account various variables such as die alignment, material size deviation, and working temperature. There is a sense of disparity between the results of FEM and the field. It is almost impossible to control all the variables of the manufacturing site. However, all factors result in forging forming load values. Theoretically, the method of calculating the forging load has already been studied^24,25^. However, in this study, forging equipment load data were used for a more practical study. By increasing the FEM result accuracy, it is possible to improve the die limit life prediction accuracy using this.

Second, as the simulation result of the forging process is essential in predicting the die life, the intervention of an expert is inevitable, and it is impossible to quickly respond to the process change. Therefore, research to improve the accuracy of the existing die life prediction method and system development research should be conducted to enable field use by non-experts.

A method to increase the prediction accuracy of die life through big data analysis by converting the variables of the working environment into data can be a solution, but the research efficiency is lowered owing to the large amount of data. There is a linking factor between the working environment variables in the cold forging process and the stress of the die, called the forming load. Therefore, in this study, the forming load data were measured in real time at the facility, and the stress acting on the die was predicted using real-time data. Consequently, it was possible to calculate the limiting life of the die with high accuracy. In addition, the data collection, processing, analysis, and monitoring procedures were integrated and systemized so that on-site workers could easily monitor the mold replacement cycle.

## Limiting life estimation of die

### Material property for part and die

**Figure 2:**
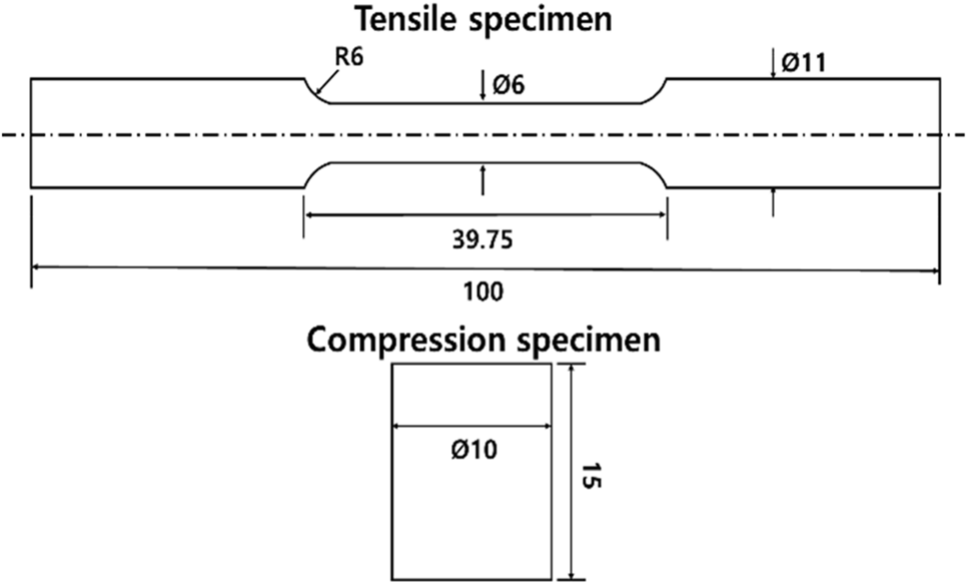
Tensile and compression test specimens with detailed dimension.

The material of the ball stud parts was 34CrMo4 (Table 1) with a diameter of 22 mm, and spheroidizing heat treatment was applied to enhance hardenability. To obtain simulation properties, tensile and compression specimens were processed, as shown in Fig. 2, in accordance with ASTM E8 (sub size) standard^26^. A tensile test was performed at a speed of 10 mm/min, and a compression test was performed at a speed of 2 mm/min up to a compression rate of 80%. As a result of the tensile test, the mechanical properties were obtained, as shown in Table 2. The engineering stress and strain obtained from the tensile and compression tests were converted to true stress and strain by the following equations.

**Table 1:** Chemical composition [wt%] of 34CrMo4.

Material	C	Mn	Mo	Si	Ni	Cr
34CrMo4	0.33–0.38	060–0.90	0.15–0.30	0.15–0.35	0.25 max	0.90–1.20

**Table 2:** Mechanical properties of 34CrMo4.

Material	Yield stress (MPa)	Tensile stress (MPa)	Uinform elogation	Total elogation
34CrMo4	420	685	0.18	0.36

1 $${\epsilon }_{true}={\int }_{{l}_{0}}^{l}\frac{dl}{l}=\mathrm{ln}(1+{\epsilon }_{eng})$$

2 $${\sigma }_{true}={\sigma }_{eng}(1+{\epsilon }_{eng})$$

**Figure 3:**
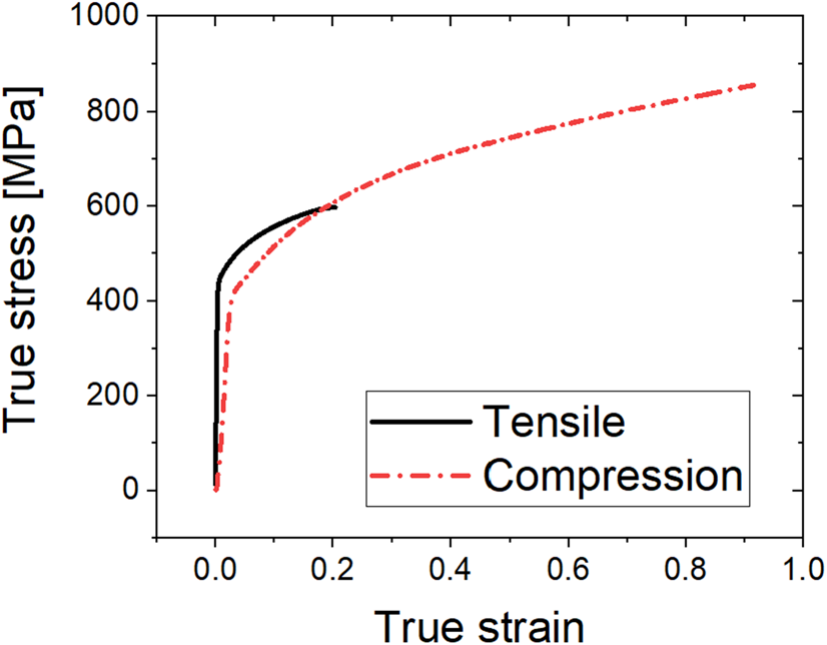
True stress–strain curve of 34CrMo4.

The tensile and compression true stress–true strain curves of the 34CrMo4 material were derived, as shown in Fig. 3. 34CrMo4 is an exclusive material for cold heading, and as a result of the tensile test, the uniform elongation section is very small, so it cannot sufficiently simulate work hardening in compression deformation.

On the other hand, in the compression test, the true stress–strain curve of a fairly wide section can be obtained because the material does not break to a compression ratio of 80%. Therefore, a compression curve was used for the simulation properties of the mulit-stage cold forging process.

**Figure 4:**
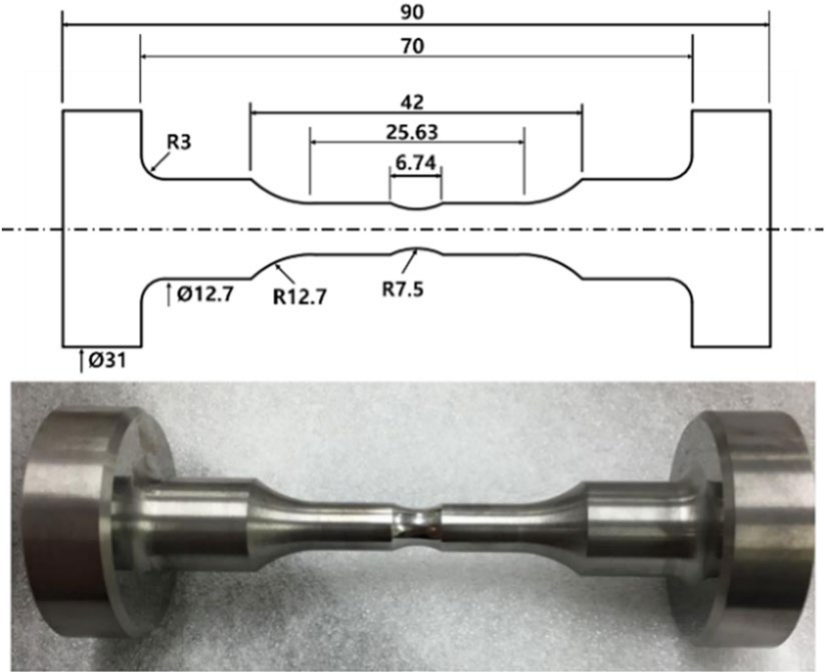
Fatigue experimental specimen with detailed dimension.

The die used in the cold forging process of the ball stud parts is generally composed of a core, reinforcing ring, and case, and the materials used are different. WC–Co alloy is used for the core, where breakage of die occurs owing to the concentration of stress in the forging process. WC exhibits high hardness and abrasion resistance, and Co is related to toughness^27^. In general, the mechanical properties of the WC–Co alloy are determined by the Co content, and it is manufactured via a sintering process of press-molding while being heated to an appropriate temperature. The material of the core die used in the manufacturing process of the ball stud parts was a WC–Co alloy with a Co content of 20%, and its mechanical properties are shown in Table 3. WC–Co alloy has high compressive strength but is vulnerable to tensile strength, so the concentration of tensile stress is suppressed by the reinforcing ring. However, when the cyclic tensile stress applied to the die material by high-speed cyclic loading exceeds a certain strength, fatigue failure occurs. Therefore, to define the limiting life of the cold forging die, it is necessary to acquire the fatigue properties of the mold material. Fatigue test specimens were manufactured through sintering, grinding, and polishing processes, as shown in Fig. 4, in accordance with the ASTM E 466 standard^28^.

**Table 3:** Mechanical properties of WC–Co.

Material	WC (wt%)	Cobalt (wt%)	Grain size	Hardness (HRA)
WC–Co	80	20	4.0–6.6	92.0

**Figure 5:**
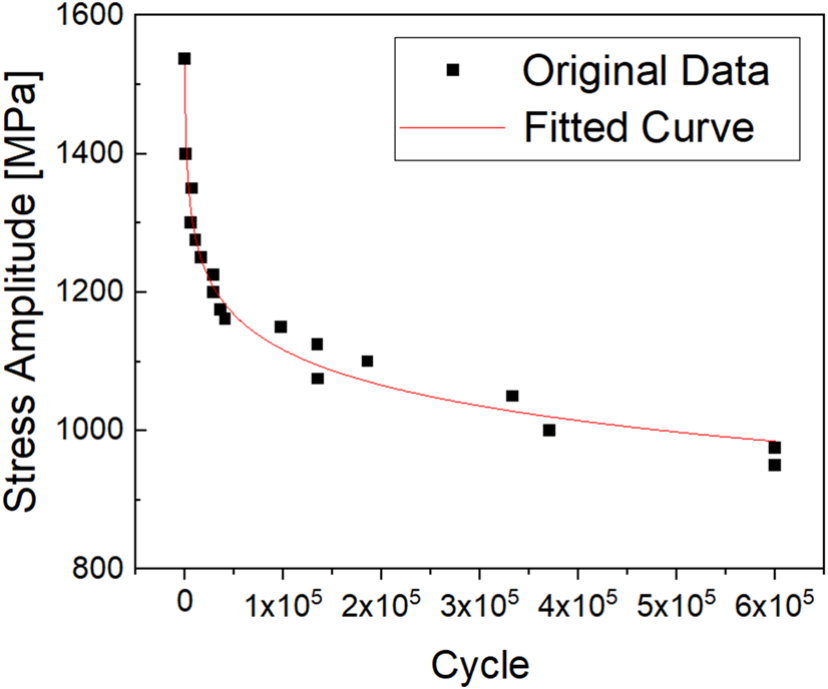
S–N curve of WC–Co material.

A radius of curvature of 3 mm was reflected to prevent stress concentration in the part in contact with the jig of the testing machine. Further, the radius of curvature of the area corresponding to the gage length was 12.7 mm, which was designed so that stress concentration could occur effectively. Using Instron 8801 equipment, the S–N diagram of the die material was derived, as shown in Fig. 5, for a stress ratio of 0.1 and a frequency of 10 Hz. Starting with the load condition corresponding to low life, the life curve progressed to the level at which the fatigue limit was secured until the flattened section.

### Simulation result of ball stud manufacturing process with die structure

**Figure 6:**
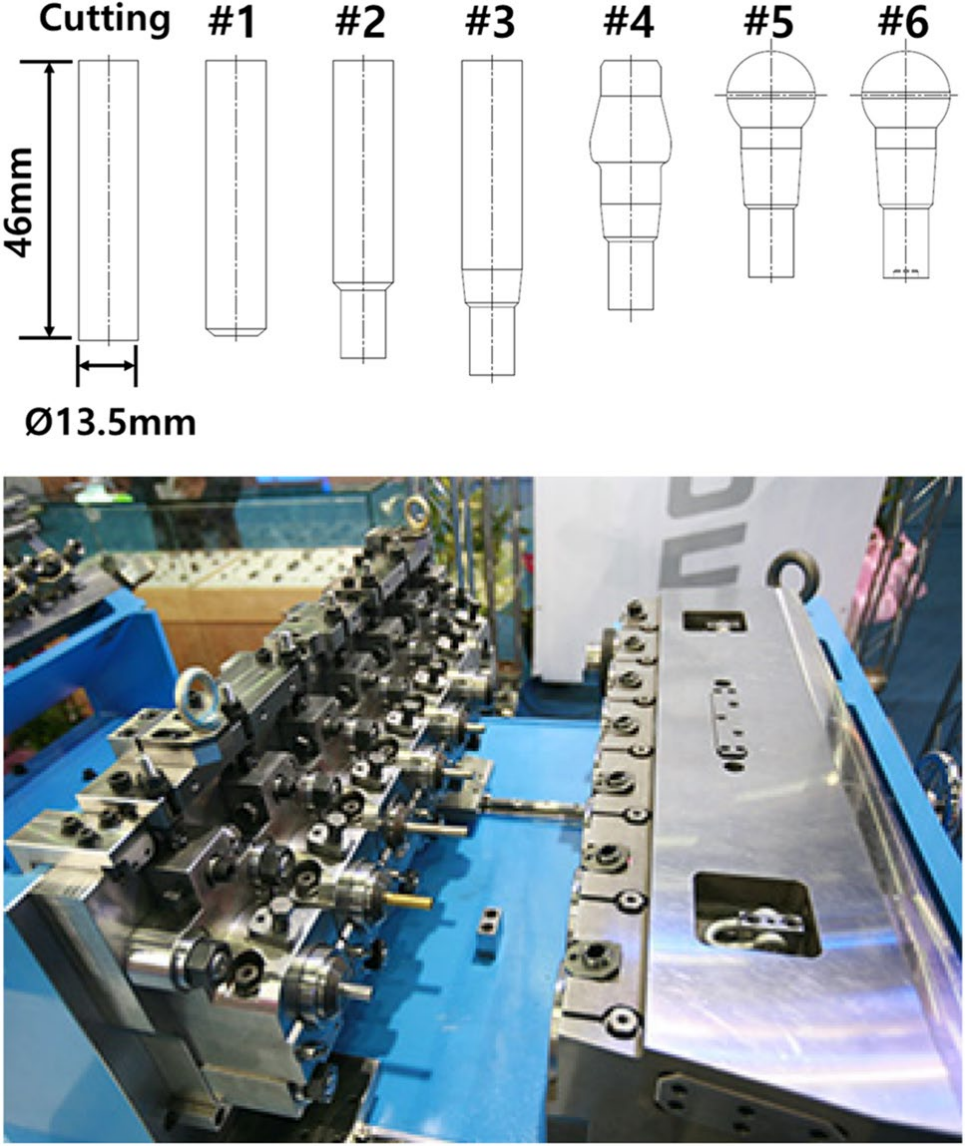
Multi-stage cold forging process design with forming apparatus.

**Figure 7:**
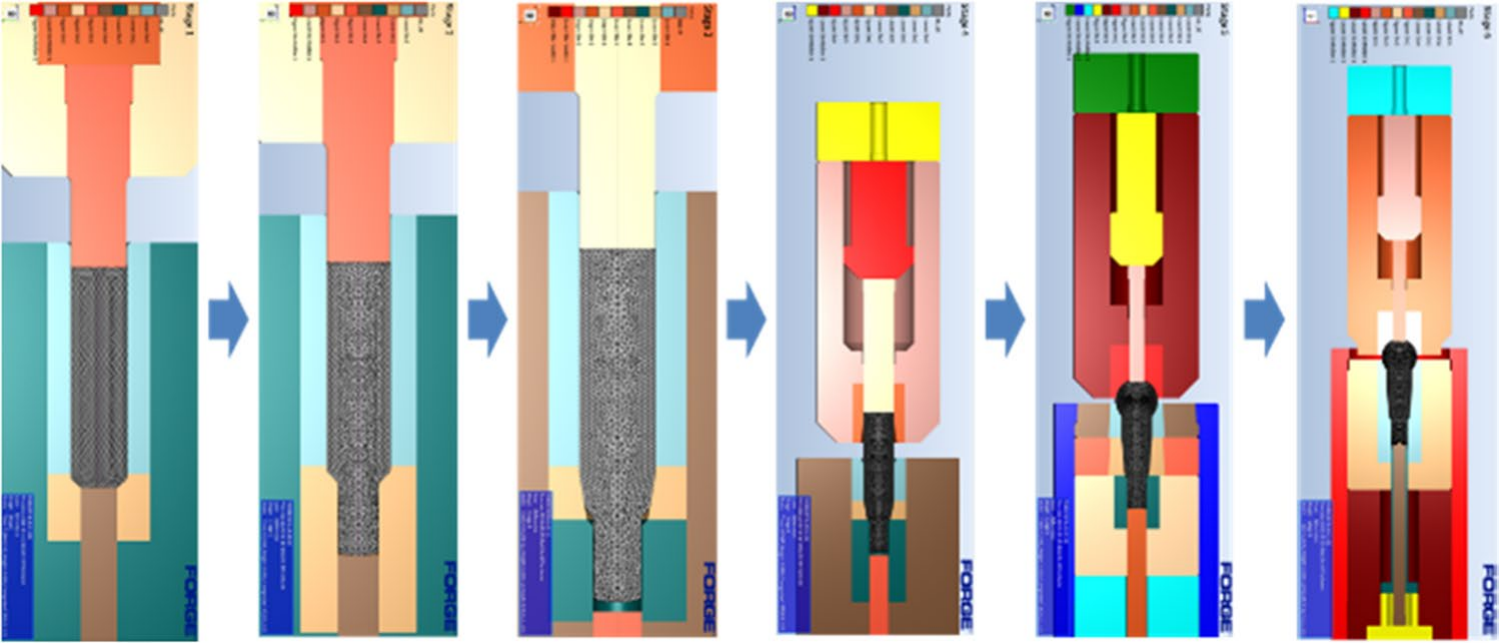
FE modeling of ball stud forming procedure.

**Figure 8:**
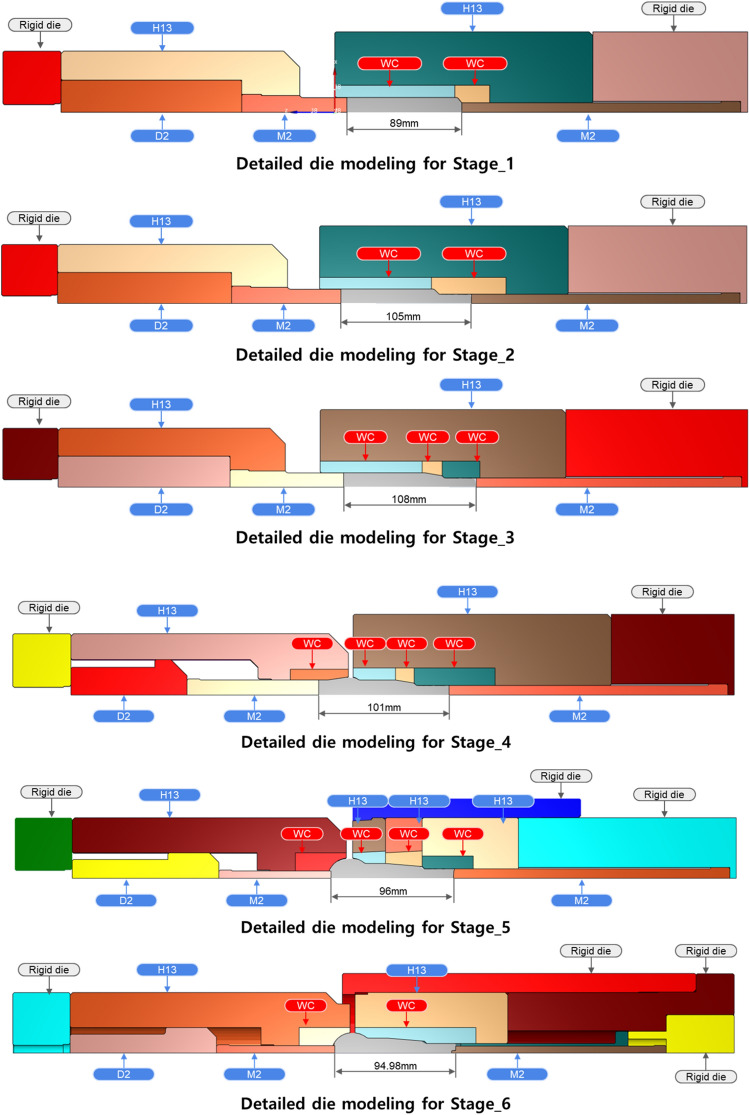
Detailed die modeling for ball stud forming procedure.

**Figure 9:**
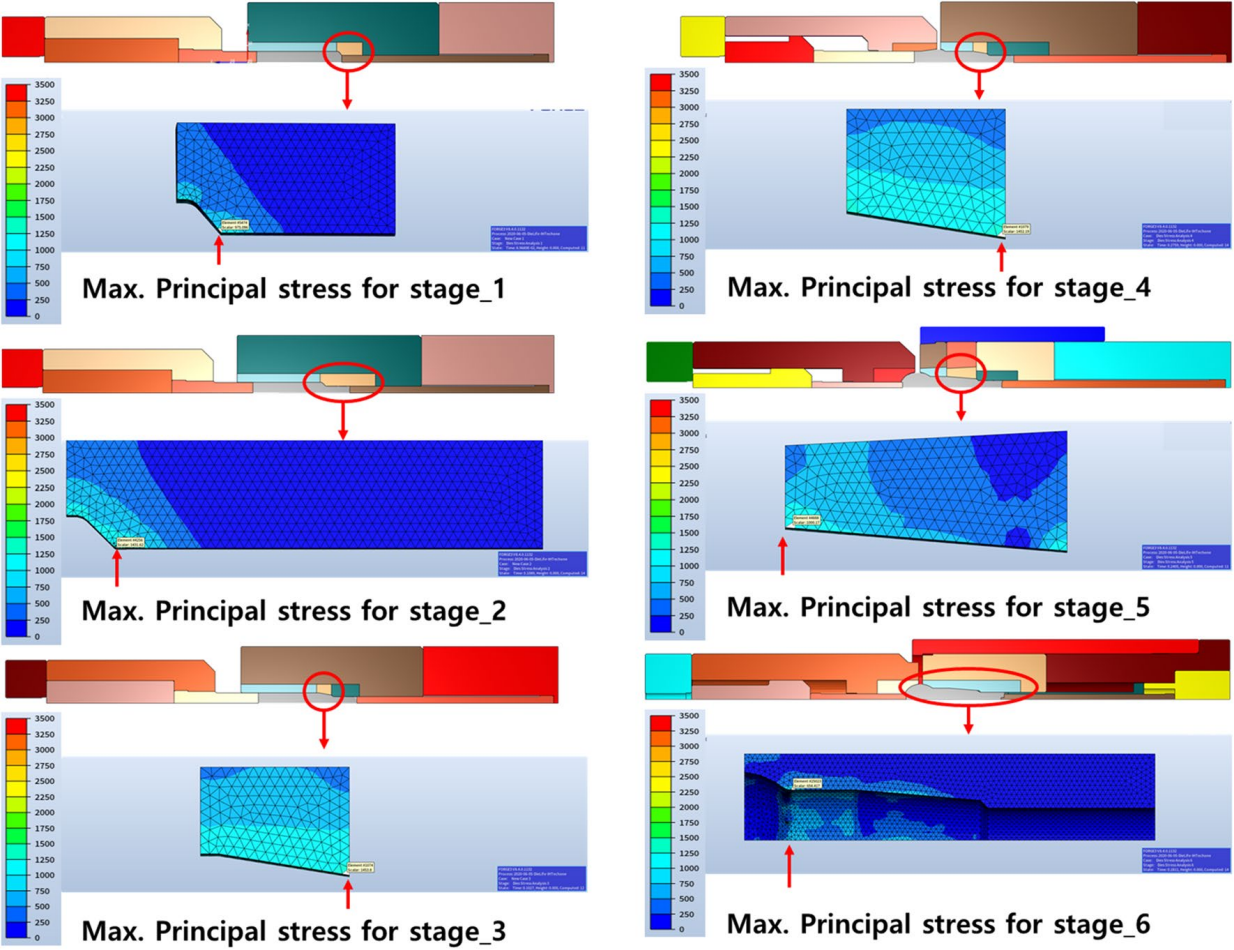
Max. principal stress acting on the core die of all stage.

The manufacturing process of the ball stud parts comprised a total of six stages with forming apparatus, as shown in Fig. 6. Different molds for each of the 6 processes are placed in one die block. After one stroke, the material automatically transfer to the next process. Accurate prediction of the tensile stress in the weak point of the core die at which tensile stress is repeatedly applied should be preceded. For this, a finite element simulation was performed on the multi-stage cold forging process using FORGE, a finite element analysis program. As shown in Fig. 7, all die structures at each stage were modeled, and a fully coupled method was applied to improve the accuracy of die stress prediction. Figure 8 shows the detailed die modeling for ball stud forming procedure. WC–Co, SKD-61, and SKD-51/SKD-11 were used for the core die (WC), reinforcement ring (H13), and case (D2/M2) material of each stage, respectively. The physical property values ​​provided by the analysis program were used as shown in Table 4. For the analysis properties of 34CrMo4, the compression diagram shown in Fig. 3 was used. The amount of shrink fit of the reinforcing ring was applied differently at each stage within the range of 0.1–0.14%. In addition, a friction coefficient of 0.08 between the material and core die was applied, and a coefficient of friction of 0.12 was applied to the rest of the contact regions. The movement speed of the punch was the same at 150 mm/s for all stages. The maximum principal stress acting on the die due to the pressurization of the material was confirmed through the fully coupled analysis. Figure 9 shows the point where the maximum principal stress in each stage acts. This analysis process is then used to derive the history of the maximum principal stress value according to the forming load in each process. The maximum principal stress value shows a constant trend according to the change of the forming load. The time it takes to confirm the results of a single analysis case is 24 h. Since it is not possible to follow the production cycle at the manufacturing site, it is simplified to the model for calculating the maximum principal stress based on the trend.

**Table 4:** Mechanic analytical properties of die materials.

Material	Young’s modulus (GPa)	Density (kg/m^3^)	Ultimate tensile strength (MPa)
WC	350	13,300	270
H13	200	7850	1990
D2	203	7860	2200
M2	207	8140	3250

### Calculation of limit die life

The maximum principal stress acting on the core die mainly occurs at the edge of the die, and this value cannot be directly substituted on the *y*-axis in Fig. 5. This is because the result value of the finite element analysis corresponds to a stress concentration dependent on the element and shape functions. Both the finite element analysis result and stress corresponding to the *y*-axis in Fig. 5 should be converted into nominal stress values. The stress concentration factor (*k*_t_) value cab be calculated based on the shape factor (corner curvature radius and depth) of the corner where breakage is expected to occur^29^. The stress concentration coefficient is a numerical value indicating the degree of stress concentration distributed in notches, holes, and grooves. By applying the stress concentration factor to the finite element analysis result value, it is possible to convert the maximum principal stress to nominal stress.

3 $${\sigma }_{analysis}=Max.\; Principal\; stress/{k}_{t}$$

Similarly, the fatigue stress concentration factor (*k*_f_) is applied to the *y*-axis stress value in Fig. 5 to convert it into nominal stress. As shown in Fig. 4, since there is a notch in the center of the specimen, the stress values are not nominal stresses. The fatigue stress concentration factor is a numerical value indicating the degree of stress concentration due to the notch in the fatigue load state.

A fatigue test specimen without a notch was additionally prepared. Under the same fatigue test conditions, *k*_f_ is calculated as the ratio of fatigue strength without notch and fatigue strength with notch.

4 $${k}_{f}={\sigma }_{without\_notch}/{\sigma }_{with\_notch}$$

Then, by dividing the stress amplitude in Fig. 5 by *k*_f_, it is converted to the nominal fatigue stress.

5 $${\sigma }_{fatigue}=stress \; amplitude/{k}_{f}$$

It is converted into nominal stress (*σ*_analysis_) by substituting the maximum principal stress, which is the analysis result, into Eq. (3). Substituting this into the nominal fatigue stress (*σ*_fatigue_) of Eq. (5), it becomes the fatigue strength that can be substituted into the S–N curve.

6 $$stress \; amplitude\_FEM={k}_{f}*{\sigma }_{analysis}$$

Quantitative evaluation of the die life was performed by predicting the life corresponding to the fatigue stress. The equation was derived by fitting the S–N diagram in Origin, a commercial S/W. By substituting the value of Eq. (6) into the fitted equation, it is possible to derive the cycle corresponding to the lifespan. The results are shown together with the actual die stress in Table 5. Comparison of the predicted data with the actual die life in the field reveals an error range of ± 20%, which is attributed to the fact that working environment variables are not taken into account in the die life prediction process. In the actual working environment, the forming load changes flexibly owing to die alignment, dispersion of material properties, and changes in friction conditions, which means that the maximum principal stress acting on the die changes according to the working environment. However, in the process of quantitatively predicting the die life, the maximum principal stress acting on the die is assumed to be an ideally fixed value, so this error is indicated. Another problem is that it inhibits applications to the field because it is difficult for non-experts to use it as the simulation of the forming process must be performed to predict the die life.

**Table 5:** Quantitative evaluation result of die life cycle.

Process	Predictive data (Cycle)	Actual data (Cycle)	Error rate (%)
1st	67,540	84,300	− 20
2nd	35,958	42,210	− 16
3rd	40,823	49,112	− 17
4th	102,796	87,099	18
5th	38,385	42,150	− 9
6th	70,067	66,087	6

## Die replacement cycle monitoring system based on actual forming load

### Real-time forming load measurement

**Figure 10:**
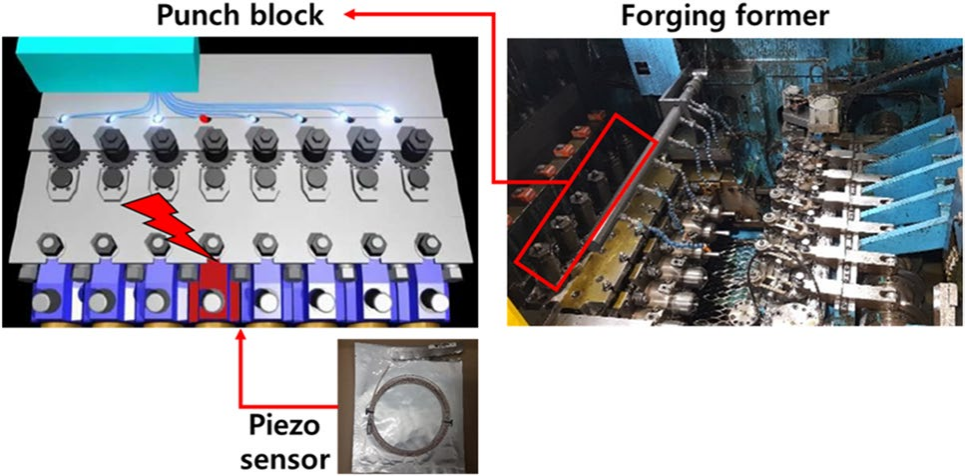
Piezo sensor installation location.

Owing to the environmental variables of the ball stud cold forging process, load variation caused by the pressurization of the upper and lower dies occurs. Accordingly, the maximum principal stress acting on the die also shows deviation. By measuring the forming load in real time, the die life prediction error can be suppressed. A piezo sensor was used to measure the pressing force of the cold forging upper and lower dies in real time. The most accurate way to measure the forming load is to install a load cell between dies. However, the load cell cannot withstand the forming load and affects the dimensional accuracy of the part. In addition, as it must be installed in an enclosed space, realizing a wired connection for signal processing is impossible. Therefore, as shown in Fig. 10, a piezo sensor was installed in the punch block of the forging former. More precisely, the piezo sensor was installed in the narrow space between the wedge and the backplate where the applied force was transmitted and can be measured. The piezoelectric sensor generates an electrical signal (*G*: gauge factor) by the piezoelectric effect^30^, which is defined as the relationship between the strain (*ε*) generated by the applied stress and the resistance rate of change (Δ*R*/*R*).

7 $$G=(\Delta R/R)/(\Delta L/L)=\Delta R/\epsilon R$$

**Figure 11:**
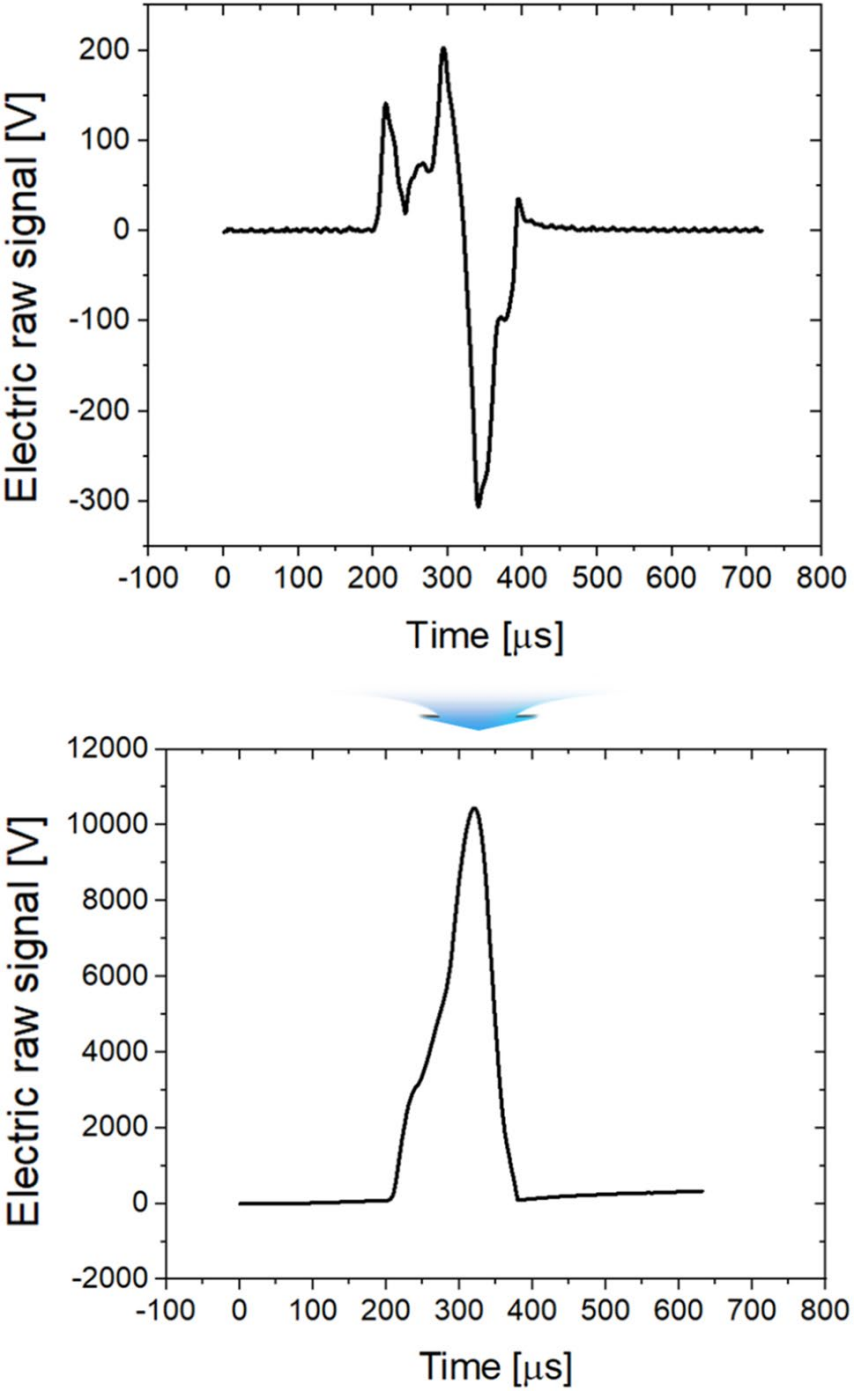
Conversion of sensor signal into load graph form.

**Figure 12:**
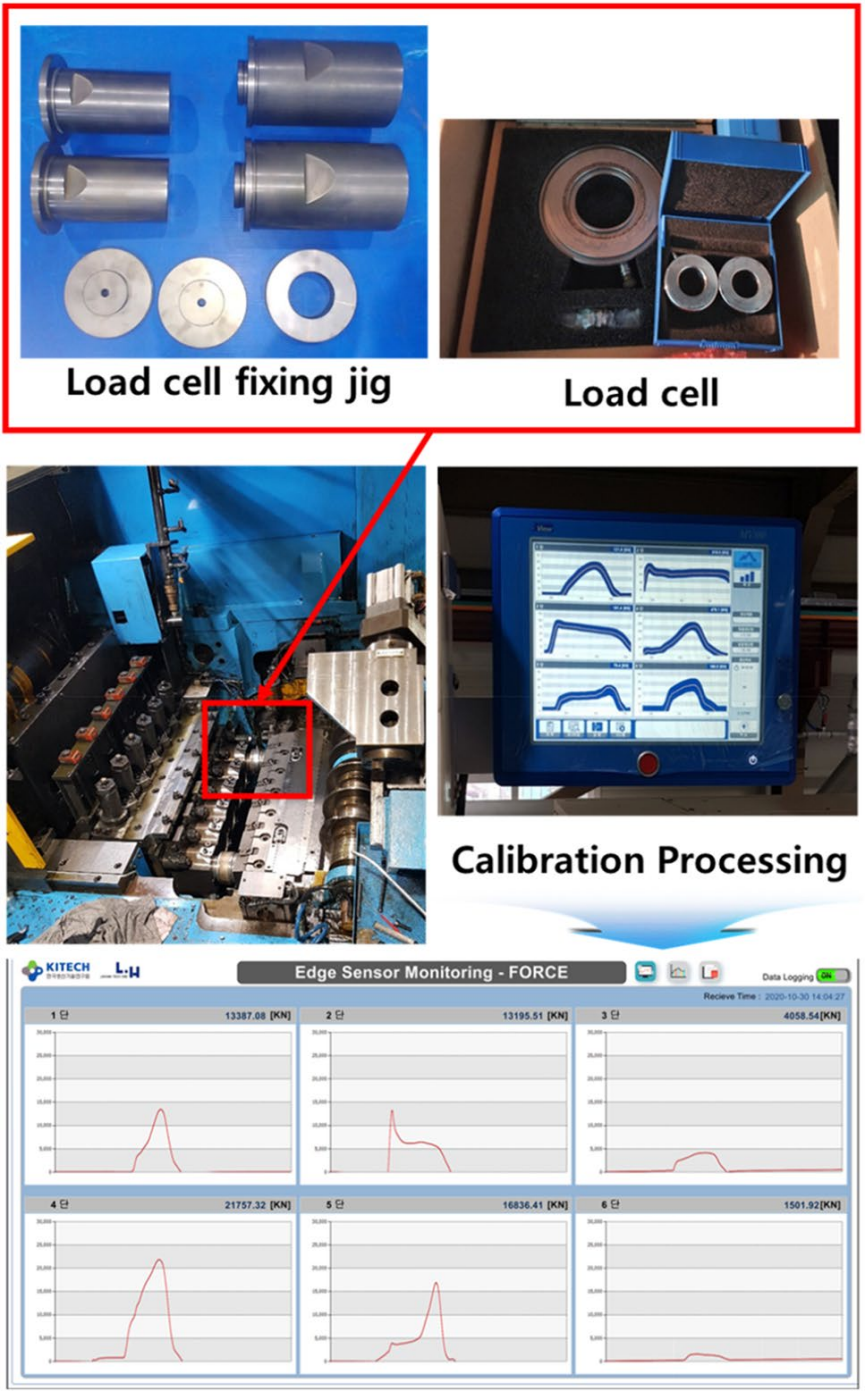
Load diagram conversion process and monitoring system.

As shown in Eq. (7), the electrical signal generated from the piezo sensor is defined as the rate of change. The electrical signal was integrated to convert it into an actual load graph, and the result is shown in Fig. 11. Finally, the electrical signal on the *y*-axis in Fig. 11 must be converted into a unit of load. To this end, a fixing jig was made to mount the calibration load cell on the forging former, and the actual load was measured. Real-time load data measurement was possible through repeated comparison of the maximum load value and maximum electrical signal value. In this process, the dedicated calibration S/W provided by the load cell vendor was used, and periodic load diagram calibration was performed on site. A system that could monitor the forming load in real time was built at the ball stud production site, and it was implemented in the form of a program, as shown in Fig. 12.

### Maximum principal stress prediction algorithm

**Figure 13:**
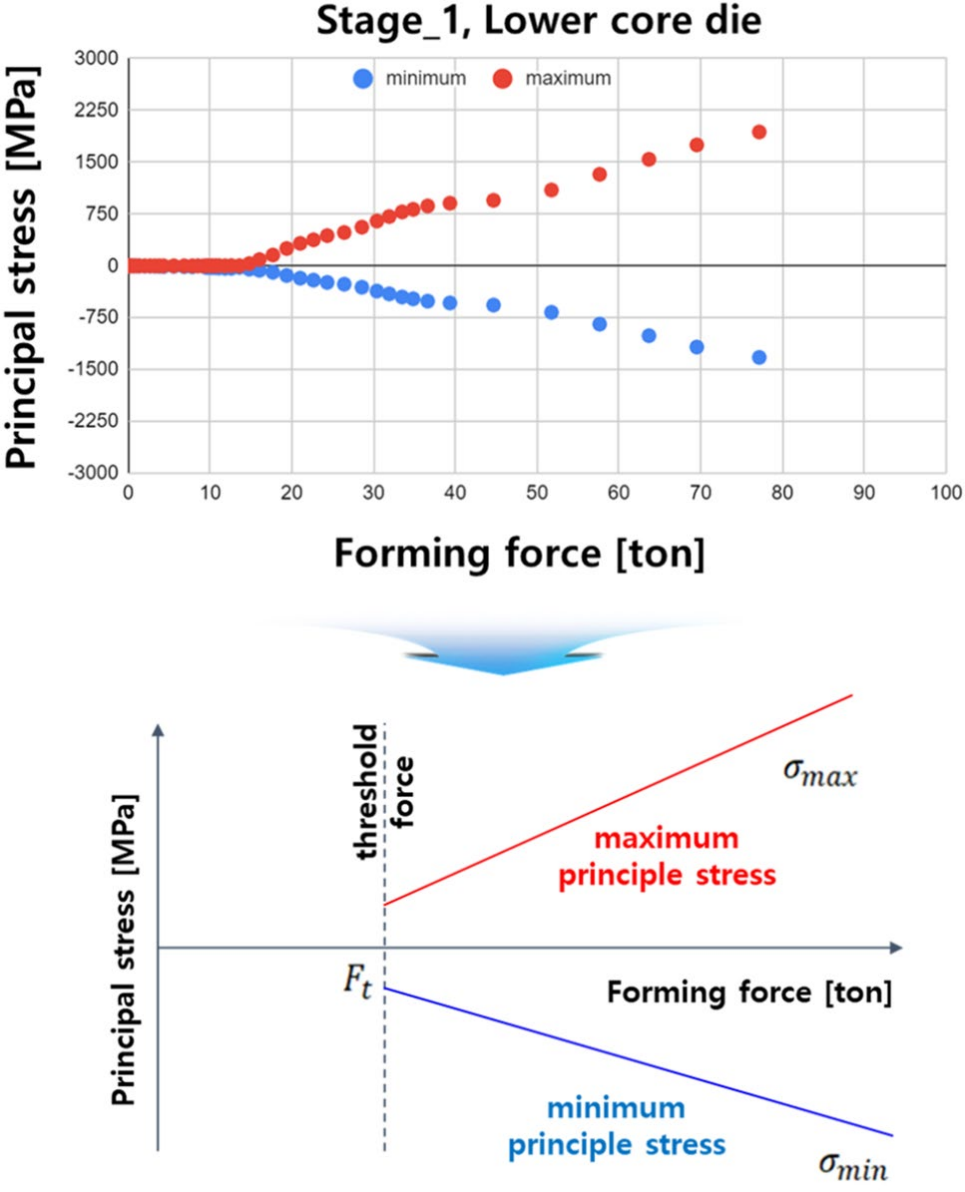
Predictive model based on maximum principle stress history.

In order for the quantitative die life prediction technology to be universally applied to the field, the maximum principal stress must also be calculated in real time based on real-time forming load data. In particular, it is impossible to perform process simulation in real time because the simulation must be carried out at high speed in consideration of the short production cycle (1ea/s) of the ball stud parts. A simple mathematical model for predicting the maximum principal stress acting on a cold forging die is a realistic alternative. Figure 13 shows the stress history at the point where the maximum principal stress occurs in the lower core die of stage 1. The maximum principal stress maintains a constant value of 0 within the range of the constant forming load (Ft: threshold load) and increases linearly and proportionally over the range of the load. This trend was also observed in the upper and lower core dies of stages 1–6. Therefore, a mathematical model capable of predicting the maximum principal stress, as shown in Eq. (8), was presented, and the C_th_ and C_slope_ constants were derived by considering the maximum principal stress history of the upper and lower dies in stages 1–6.

8 $${\sigma }_{max}={C}_{th}+{C}_{slope}{F}_{real}$$

**Figure 14:**
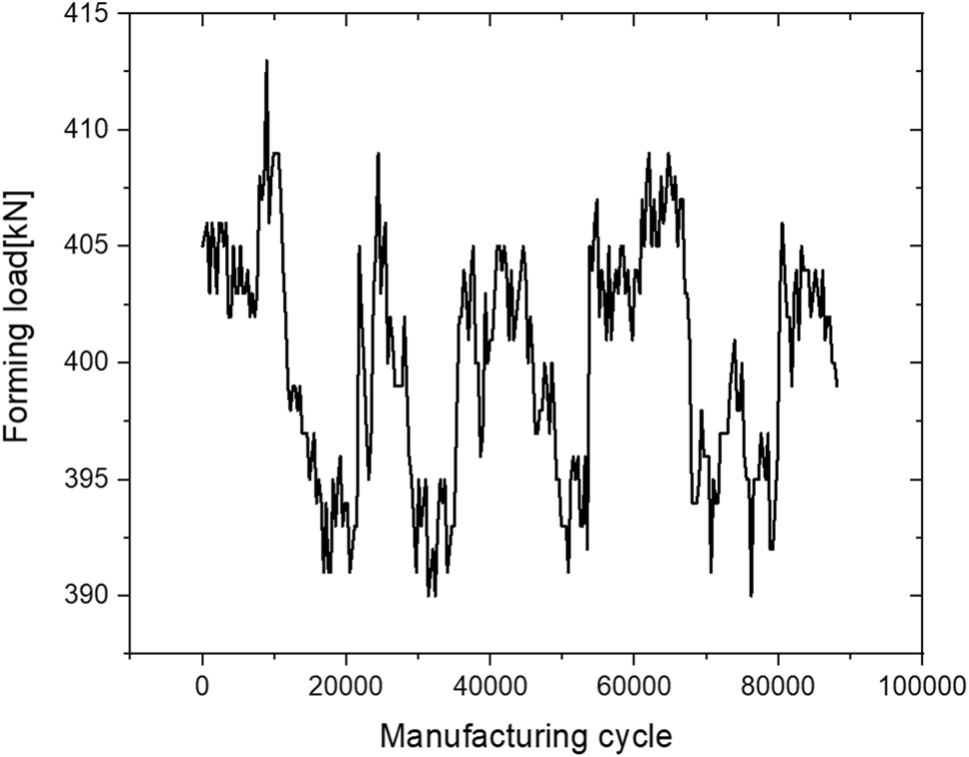
Real time maximum forming load history.

**Figure 15:**
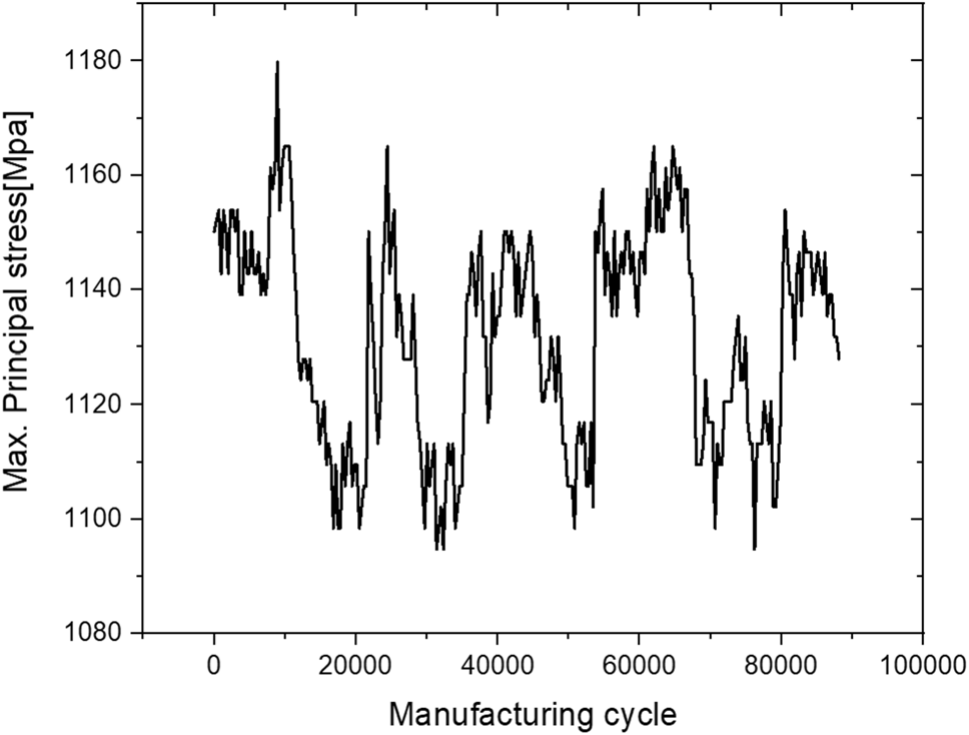
Real time maximum principal stress history.

*F*_real_ is shown in Fig. 14 and denotes the maximum forming load value from stage 1 to stage 6 measured in real time. Its conversion into the maximum principal stress history using Eq. (8) is shown in Fig. 15. Such complex variable load histories can be replaced with simplified equivalent load histories; however, in this study, actual data were used for the real-time implementation and simplification of field application algorithms.

### Die life prediction based on linear cumulative damage

Miner's linear cumulative damage hypothesis was derived under the assumption that the fracture of the structure due to fatigue occurs when the work caused by countless fatigue loads reaches the critical value of the material^31^. Using the maximum principle stress data and the S–N diagram of the material, it is possible to calculate the cumulative damage factor (CDF), as in Eq. (9)@^32^.

9 $${\mathrm{DF}}_{i}=\frac{{n}_{i}}{{N}_{i}}, CDF=\sum {\mathrm{DF}}_{i}$$

**Figure 16:**
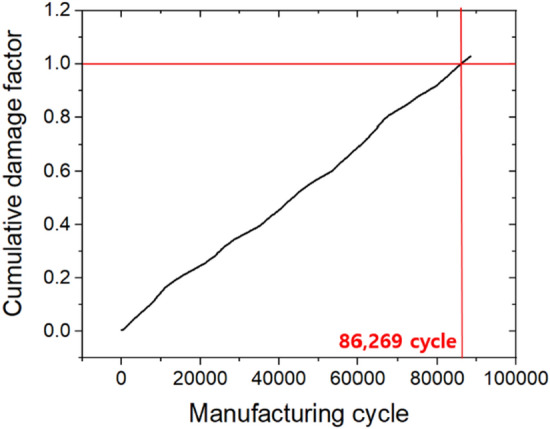
Die life cycle prediction based on cumulative damage factor.

Here, *n*_i_ is the number of cycles according to each stress level, *N*_i_ is the allowable number of cycles according to the stress criterion obtained from the fatigue curve, and *DF*_i_ is defined by the relationship of *n*_i_/*N*_i_. As shown in Fig. 16, the cycle in which the value of CDF reaches 1 by cumulative calculation of DF, was defined as the limiting life of the forging die. Table 6 shows the life limit for each process. It can be seen that the error range was reduced to ± 7% compared to the die life prediction results in Table 5, assuming the maximum principal stress value to be a single constant.

**Table 6:** Quantitative evaluation result based on real-time forming load data.

Process	Predictive data (Cycle)	Actual data (Cycle)	Error rate (%)
1st	86,269	84,300	2.3
2nd	45,075	42,210	6.8
3rd	47,675	49,112	− 2.9
4th	88,413	87,099	1.5
5th	41,300	42,150	− 2.0
6th	65,967	66,087	− 0.18

### Die life managing system

**Figure 17:**
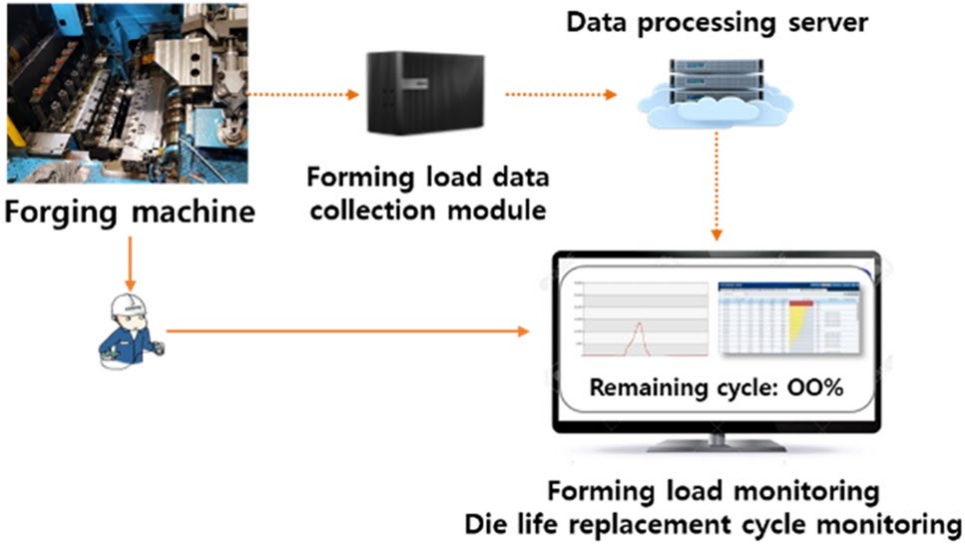
Die life managing system.

The system is configured as shown in Fig. 17 so that the operator can monitor the remaining life cycle of the die currently in use. The forging machine has an embedded piezo sensor, and a module to store the signal generated by the sensor is installed. In addition, a data processing server for calculating the sensor signal as the remaining life cycle of the die is configured. The remaining life cycle of the die can be calculated as in Eq. (10).

10 $$\left(1-\mathrm{CDF}\right)\times 100 [\mathrm{\%}]$$

## Conclusions

In this study, a method to more efficiently manage the die life in the multi-stage cold forging process was presented. Based on pressurized load data collected in real time, a more accurate prediction of die life was possible. Furthermore, to increase the utilization in the field, the intervention of experts was completely excluded, and application to the automobile parts manufacturing site was realized. The detailed study contents are summarized as follows.

1. The S–N diagram of the die material was obtained to predict the lifespan of the cold forging die. The maximum principal stress value was predicted through the coupling of the forging process simulation and die analysis. It was possible to predict the die life by substituting the maximum principal stress value into the S–N diagram, but the accuracy was low with an error rate range of ± 20%. In addition, there was a limit to field application owing to the high professional difficulty of the die life prediction process.
2. To solve this problem, an infrastructure for real-time monitoring of the forming loads from stages 1–6 of cold forging process was established. In addition, a data sensing–collection–analysis–processing linkage system was installed at the manufacturing site so that the forming load data could be used to predict the life of the die. To exclude expert intervention in this process, a mathematical model capable of predicting the maximum principal stress based on the forming load data was presented.
3. As a result of predicting the die life more accurately based on the linear cumulative damage hypothesis, the error range was reduced from a maximum of ± 20% to ± 7%.
4. With the establishment of a system capable of monitoring the remaining life of the die, the operator in the field can intuitively determine the time to change the die, and it is possible to improve the efficiency of the manufacturing process.
